# The Herpetofauna Present in the Province of Pastaza in Ecuador: Diversity and Conservation Status

**DOI:** 10.3390/biology15060451

**Published:** 2026-03-10

**Authors:** Cinthya Garcia-Romero, Sarah Martin-Solano, Paola Araujo-Erazo, Alexandra D. Hernández Hernández, Santiago Paredes, Andrés Prado-Aguas, Gabriel Carrillo-Bilbao

**Affiliations:** 1 Carrera de Educación Básica Presencial, Facultad de Filosofía, Letras y Ciencias de la Educación Universidad Central del Ecuador, Quito 170521, Ecuador; cagarciar@uce.edu.ec; 2 Instituto de Investigación en Zoonosis, Universidad Central del Ecuador, Quito 170521, Ecuador; ssmartin@espe.edu.ec; 3 Laboratorio de Biotecnología Animal, Grupo de Investigación en Sanidad Animal y Humana (GISAH), Carrera Ingeniería en Biotecnología, Departamento de Ciencias de la Vida y la Agricultura, Universidad de las Fuerzas Armadas-ESPE, Sangolquí 171103, Ecuador; 4 Carrera de Pedagogía de las Ciencias Experimentales, Química y Biología, Universidad Central del Ecuador, Quito 170521, Ecuadoralehernandez2301@gmail.com (A.D.H.H.);; 5 Instituto Nacional de Biodiversidad, Quito 170135, Ecuador; 6 Red Ecuatoriana de Universidades para Investigación y Posgrados, Quito 170521, Ecuador

**Keywords:** alpha diversity, reptiles, amphibians, *Atelopus spumarius*

## Abstract

The province of Pastaza is a biodiversity hotspot in the Amazon but lacks up-to-date inventories of its herpetofauna. This study aimed to provide an updated characterization of the richness, composition and conservation status of amphibians and reptiles in different habitats. Between 2013 and 2018, standardized sampling was carried out using quadrats (8 × 8 m) and transects (100 × 20 m), as well as pitfall traps and daytime and night-time visual censuses. A total of 900 h of effort was distributed across six habitats. Coverage estimators, species accumulation curves, range abundance curves, and alpha diversity indices (Shannon, Simpson, and Chao-1) were used for data analysis, as well as similarity indices (Jaccard). A total of 75 species were recorded (51 anurans, eight lizards, 14 snakes and two salamanders), displaying habitat-dependent dominance patterns led by *Dendropsophus sarayacuensis* and *Scinax ruber*. Diversity is considered high according to the Chao-1 (19.63–60.53), Shannon–Weaver (2.402–3.223), and Simpson (0.861–0.947) indices, showing interannual variation with a temporary decrease in year 5 and an increase in year 6. The presence of species at risk (VU, *n* = 26 spp. and EN, *n* = 2) and high temporal variability highlight the need for continuous monitoring and conservation strategies tailored to specific habitats.

## Simple Summary

Pastaza, a highly biodiverse area of the Amazon rainforest, lacks recent information about its amphibian and reptile species. Between 2013 and 2018, this study surveyed different habitats using several search methods, both day and night. The researchers found 75 species, including frogs, lizards, snakes and salamanders. Some species were much more common depending on the habitat. Overall diversity was high, but some species are at risk and communities changed over time. These findings highlight the importance of ongoing monitoring and conservation efforts tailored to each habitat type.

## 1. Introduction

Amphibians and reptiles are characterized as the most diverse and abundant group of vertebrates in tropical ecosystems [1]. There are currently 8986 species of amphibians [2] and 12,502 reptiles worldwide [3], of which 41% of amphibians and 21.1% of reptiles are endangered, with 37 and 31 respectively having become extinct since 1500 [4,5,6].

Amphibians and reptiles are an example of highly threatened taxa with around 2873 species classified as threatened, mainly in the Caribbean islands, Mesoamerica, the tropical Andes, the mountains and forests of western Cameroon and eastern Nigeria, Madagascar, the Western Ghats, and Sri Lanka [5]. In the case of reptiles, 1829 threatened species are reported, concentrated in Southeast Asia, West Africa, northern Madagascar, the northern Andes, and the Caribbean. However, the limited information available creates uncertainty about the status of species with insufficient data, as it ranges from 18.0% (assuming that no species with insufficient data is threatened) to 32.8% (assuming that all species with insufficient data are threatened) [4].

Currently, the status of amphibians and reptiles is deteriorating worldwide, especially in the Neotropics [7,8], due to various factors such as habitat loss and modification [9], invasive species [10], emerging diseases [11], pollutants [12], trade [13], and climate change [14,15,16,17,18]. In Latin America, the situation is similar, even in biodiversity hotspots that are home to exceptional concentrations of species [19], where rapid losses of natural habitat are causing amphibians to face serious threats of extinction in areas such as Central America [15,20], South America [18,21], Europe [22,23,24], and Asia [25].

According to Cortéz-Gómez et al. [26], 76% of studies based in South America determined several of the ecological functions of amphibians, including the nutrient cycle [27], energy flow through food chains, as predators [28] and prey [29], seed dispersal [26], and pollination [30]. Notably, the importance of amphibians can also be seen in aspects such as culture [31], medicine [32], education and research [33], tourism, and food [34]. Given that one of the common characteristics of amphibians is their glandular, smooth, and permeable skin, which requires high levels of humidity, they are considered bioindicators of environmental health, mainly in aquatic environments [33].

Reptiles play important roles in natural systems, as do amphibians, mainly as bioindicators of environmental health [35], due to their associations with microhabitats [36], the role they play within the food chain [26], and the connection between aquatic and terrestrial ecosystems [37]. Considering that knowledge about reptile diversity and species identification remains scarce, the decline in their populations is associated with biological characteristics and environmental factors [38,39].

Ecuador is one of the world’s megadiverse countries, ranking third in terms of reptile species with 512 species and third in terms of amphibian species with 676 species [40]. However, anthropogenic activities in Ecuador have led to the loss and transformation of habitats and, in turn, to the decline of animal and plant species. As a result, amphibians are highly threatened due to their direct dependence on humidity and temperature [5]. In Ecuador, the greatest richness of species is concentrated in the most diverse Amazonian provinces such as Napo, Sucumbíos, and Pastaza, which, due to their biogeographical characteristics, allow for high diversification and endemism within the largest expanses of natural ecosystems at the regional level. This region is recognized as a priority area for national and global conservation [41].

This study aims to assess the species richness and composition of the herpetofauna at the Fátima Amazonian Scientific Station (ECAF), thereby providing updated information on the diversity of amphibians and reptiles in Pastaza Province. Furthermore, by documenting species alongside their IUCN Red List threat categories, the study will provide baseline data to support the development and prioritization of conservation strategies within the province.

## 2. Materials and Methods

### 2.1. Study Area

**Figure 1:**
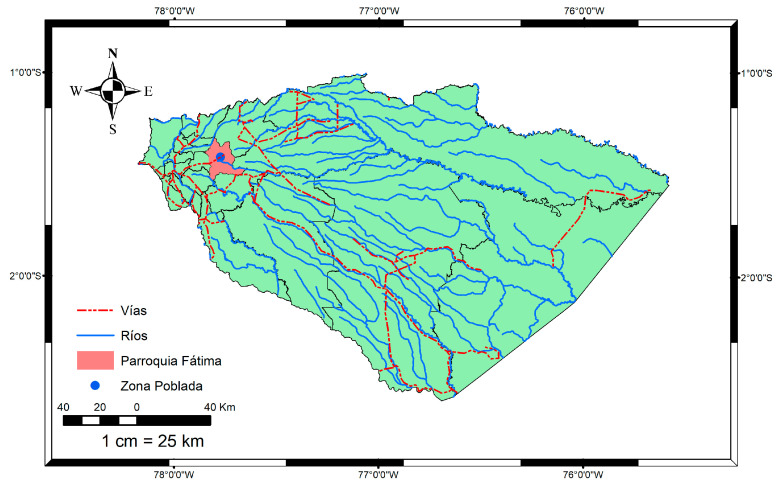
Sampling map of herpetofauna in the Pastaza province.

The research was conducted in the parish of Fátima, located in the central Amazon region of the province of Pastaza, in southeastern Ecuador (Figure 1). It has an altitude gradient ranging from 900 to 1100 m above sea level. The average temperature in Pastaza is 21.60 °C, according to the annual bulletins of the WWO (World Weather Online, https://map.worldweatheronline.com/, accessed on 20 December 2025).

### 2.2. Monitoring

**Figure 2:**
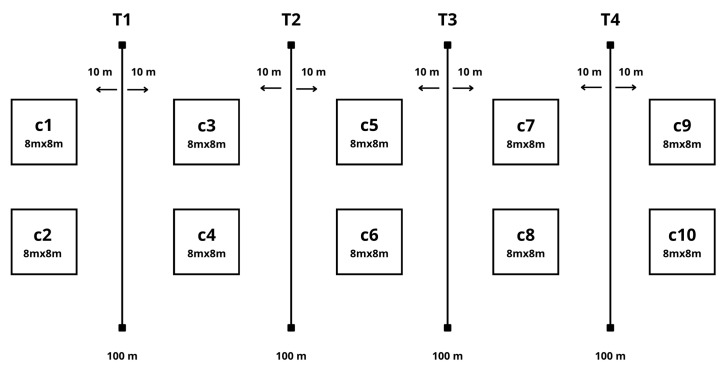
Sampling design showing four linear transects (T1–T4), and ten quadrats (C1–C10) used for amphibian and reptile surveys across six habitat types.

To conduct the study, we followed the Guidelines For Use Of Live Amphibians And Reptiles In Field and Laboratory Research [42] and the suggestions of Gray et al. [43] to ensure biosecurity measures and cause minimal impact on amphibians and reptiles. The inventory and monitoring techniques were applied to plots or quadrants, fixed-band transects, and random visual encounters [44,45,46]. Ten 8 m × 8 m quadrants, four 100 m × 20 m linear transects, and 50 passive pitfall traps were set up randomly in six types of habitats: swamp (forest), swamp (grassland), ECAF facilities, primary forest, disturbed forest, and stream-slope (Figure 2).

The inventory was carried out between September 2013 and September 2018. During this time, visual and photographic records of amphibians and reptiles were obtained through direct daytime and night-time searches (five hours each) within the six habitats. In total, the sampling effort comprised six years of 15 days each, amounting to 900 h of surveys per biotope, divided in trimestral study period 50 h to consider the cycle of species and their variability during every year.

Habitats were classified in order to represent the environmental heterogeneity of the study area and capture the ecological variability relevant to herpetofauna. Six habitat types were defined based on structural and ecological criteria, including vegetation physiognomy and stratification, degree of anthropogenic disturbance, presence and type of aquatic environments, and associated geomorphological characteristics. The habitats identified were: wooded swamp, herbaceous swamp, primary forest, disturbed forest, stream slopes, and areas altered by infrastructure (ECAF facilities). These environmental units represent contrasting conditions that determine the availability of microhabitats, refuges, and trophic resources, directly influencing the composition and distribution of amphibians and reptiles.

### 2.3. Species Identification

The recorded species were identified through a combined field-based and literature-supported approach. Initial identification was performed in situ using external morphological characteristics such as coloration patterns, body size, skin texture, and other diagnostic traits observable in the field. These identifications were subsequently corroborated by reviewing information compiled from Field Guides to Amphibians and Reptiles of Ecuador [47,48], databases available at the Field Museum [49], and amphibian and reptile photographic plates available on the BioWeb platform of the Pontifical Catholic University of Ecuador [41]. Given the high taxonomic complexity and morphological similarity within certain groups, particularly the genus Pristimantis, and in the absence of molecular analyses, individuals belonging to this genus were conservatively identified only at the genus level (*Pristimantis* sp.) [50].

The conservation status matrix was compiled using field data that included species presence/absence, relative abundance (number of records per species), habitat type, day and time of observation, and the location where each species was recorded; these data were subsequently linked to the corresponding IUCN conservation categories, which was obtained from the Red List of Threatened Species of the International Union for Conservation of Nature [6]. This list classifies species according to their risk of extinction and global conservation status.

### 2.4. Statistical Analysis

#### 2.4.1. GCE (Global Coverage Estimator)

The global coverage estimator (GCE) was interpreted based on the improved formula of Chao and Jost [51] sample estimator which quantifies the proportion of the total abundance of individuals in a community. This estimator is a direct measure based on information about the rarest species. Its calculation is derived from the following formula:

(1) $$\hat{C}=1-\frac{F_{1}}{N}\;\left(\frac{F_{1}-1}{F_{1}-1+2F_{2}}\right)$$

where

$\hat{C}$ = estimated sample coverage

*N* = total number of individuals sampled (the sum of the abundances of all recorded species).

$F_{1}$ = number of singleton species (species represented by exactly 1 individual)

$F_{2}$ = number of doubleton species (species represented by exactly 2 individuals)

A high value (100%) indicates that there is a low probability that the next individual captured will belong to a species already recorded. This calculation was applied to the abundance matrix to obtain the overall value and was disaggregated to evaluate the sampling effort in each habitat (swamp (forest), swamp (grassland), facilities, primary forest, disturbed forest, and stream-slope).

#### 2.4.2. Rank Abundance Curve

A standardized sampling protocol was implemented with fixed times, periods, and locations the estimation for abundance curves across habitat types. Sampling effort and the number of individuals recorded per sampling unit were systematically quantified. The data were then organized by habitat and the relative abundance of each species within each habitat (swamp forest, swamp grassland, facilities, primary forest, disturbed forest and stream slope) was calculated. The data were then organized from highest to lowest abundance and the corresponding curve was constructed using these values. This graphical representation was generated using PAST 5.2 software [52], which enabled us to visualize patterns of species richness in the different habitats. This procedure enabled us to compare the structure of herpetofauna communities and demonstrate how species composition and abundance vary according to the ecological characteristics of each habitat type.

#### 2.4.3. Alpha Diversity

Alpha diversity was assessed using cumulative species records and relative abundance within standardized sampling units, focusing on community-level patterns rather than estimates of population size or density. Diversity was estimated from the general species database by calculating three indices [52] that characterize community structure. The Chao1 index [53] was used as an estimator of richness, incorporating the frequency of rare species associated with sampling. Shannon-Weaver diversity [54] was calculated considering both richness and relative abundance through species distribution analysis. Finally, the Simpson index [55] was used to quantify dominance within the community, giving greater weight to the most abundant species and allowing the identification of possible patterns of concentration. These indices provide a comprehensive assessment of alpha diversity and facilitate comparison between sampling units.

#### 2.4.4. General Species Accumulation Curve

The species accumulation curve was generated in the PAST software program [52] using a presence matrix for each sampling unit. The program performed random permutations of the data to calculate the expected cumulative richness as the number of specimens increased. This produced the average curve (red line), along with its confidence interval (shaded area). This procedure provided a robust estimate of the recorded richness and enabled a visual assessment of the adequacy of the sampling effort to be made.

#### 2.4.5. Species Similarity: Jaccard Index

To evaluate temporal variation in species composition, a presence-absence matrix was constructed for each sampling year, considering the total number of species recorded in each year. The Jaccard index, defined as the proportion of shared species relative to the total number of species observed in each comparison, was then used to calculate the similarity between years from this matrix. These values were then transformed into distances to enable hierarchical clustering analysis. The average linkage method (UPGMA) was then applied to generate a dendrogram, visualizing the affinity and degree of species replacement between years. The dendrogram structure obtained reflected clustering patterns between periods with greater similarity in composition, facilitating identification of temporal changes in the herpetofauna community.

### 2.5. IUCN Species Conservation Status

The conservation status matrix was compiled from records obtained during field monitoring. Each individual was identified using specialized herpetofauna guides and reliable online taxonomic sources. Once the identity of each species had been confirmed, its threat category was verified using the official IUCN database. The records were organized into a matrix that summarized the number of individuals per species and their corresponding conservation category. This allowed for the visualization of the distribution of sampling effort and the degree of threat associated with each taxon. Finally, it should be noted that there are species in the Not Evaluated (NE) category because individuals could only be identified to genus level.

## 3. Results

A total of 614 individuals were observed during the field survey. Of these, 551 individuals belonged to the order Anura, comprising 51 species, 18 genera, and six families (Aromobatidae, Bufonidae, Dendrobatidae, Hylidae, Leptodactylidae, and Strabomantidae). A further 39 individuals belonging to the order Squamata (lizards) were recorded, comprising eight species, five genera, and three families (Anolidae, Gymnophthalmidae, and Sphaerodactylidae). Additionally, 22 individuals belonging to the order Squamata (snakes) were recorded, comprising 14 species, 10 genera, and five families (Boidae, Colubridae, Dipsadinae, Elapidae, and Viperidae). Finally, two individuals belonging to the order Caudata (salamanders) were recorded, comprising two species, one genus, and one family (Plethodontidae).

### 3.1. GCE (Global Coverage Estimates)

On a global scale, the GCE (97.4%) indicates a high number of species recorded in the study area, demonstrating its biodiversity. Homogeneous sampling was achieved across the evaluated habitats, with values ranging from 93% to 100% (Table 1). However, slight variations exist between habitats: ECAF facilities coverage is slightly lower at 93.8%, while swamp forest and stream slope coverage is for both habitats very high at 100%, which favors the detection of species associated with riparian systems. Primary forests also have high coverage, ensuring adequate inclusion of generalist and synanthropic species.

### 3.2. Abundance

Abundance patterns were assessed using the number of individuals recorded per species within standardized sampling units. These values represent counts of individuals and were used to construct rank–abundance curves. Species similarity was assessed by integrating records from the entire six-year sampling period (rank) and comparing herpetofaunal assemblages among habitat types, thereby minimizing the effect of interannual variability. The location with the highest abundance of individuals was primary forest with 250 individuals.

**Figure 3:**
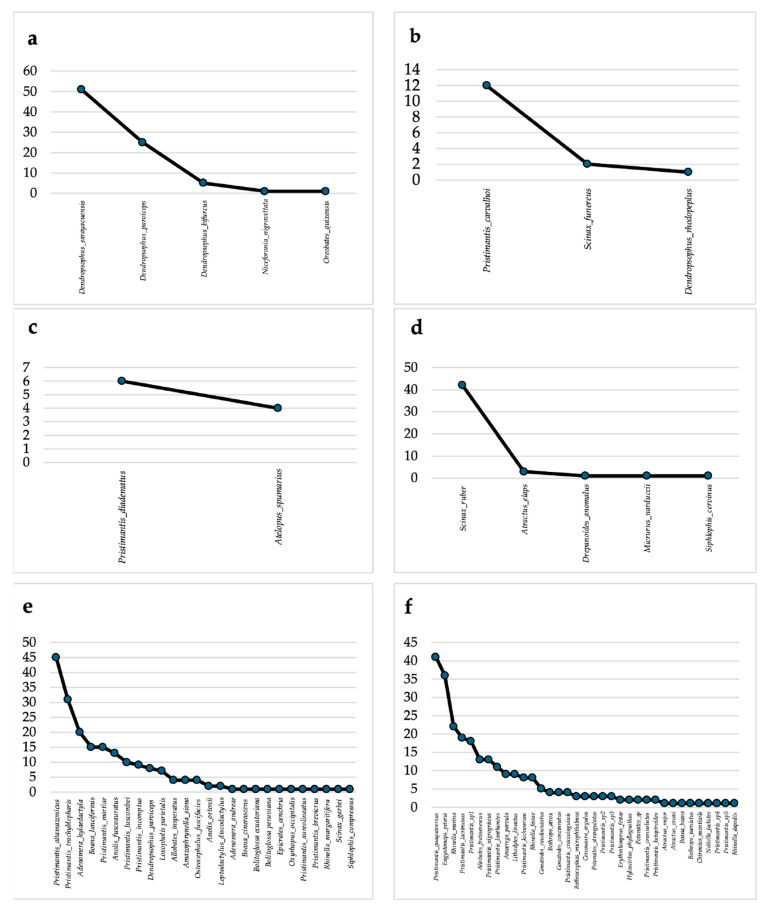
Rank abundance curve. (**a**) Swamp (grassland). (**b**) Swamp (forest). (**c**) Stream-slope. (**d**) ECAF Facilities. (**e**) Primary forest. (**f**) Disturbed forest.

The abundance in the riparian habitats was variable. The abundance graph for the swamp (grassland) (Figure 3a) shows a higher incidence of *Dendropsophus sarayacuensis* (*n* = 51), which had the highest number of individuals and acted as the dominant species in this habitat. However, the abundance graph for the swamp forest (Figure 3b) showed a community dominated by *Pristimantis carvalhoi* (*n* = 12), followed by *Scinax funereus*, which is significantly less abundant (*n* = 2). The abundance graph for the stream slope (Figure 3c) indicates a herpetofauna community with low species richness (*n* = 2), the dominant species is *Pristimantis diadematus* (*n* = 6), followed by *Atelopus spumarius* (*n* = 4).

The abundance graph for the primary forest (Figure 3e) shows the most diverse herpetofauna community. The most dominant species in this community are *Pristimantis altamazonicas*, followed by *Pristimantis trachyblepharis* and *Adenomera hylaeodactyla*. This dominance underscores the significance of leaf litter and undergrowth in this habitat. Although abundance declines, records of snake, lizard and other amphibian species are maintained.

In the case of ECAF facilities, the abundance graph (Figure 3d) showed a community dominated by the tree-dwelling species *Scinax ruber* (*n* = 42). The abundance graph for the disturbed forest (Figure 3f) revealed a community with a high concentration of individuals led by *Pristimantis quaquaversus*, followed by *Rhinella marina*.

**Figure 4:**
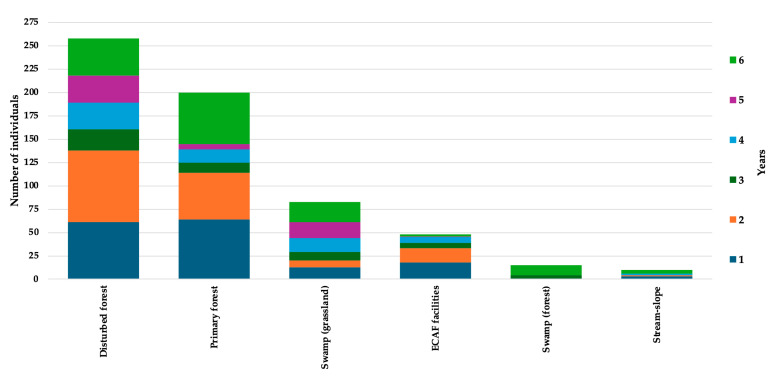
Total number of individuals recorded across six habitat types over the study period, showing yearly variation in abundance (Years 1–6).

The annual record of individuals by habitat type shows a clear dominance of forested environments throughout the study period (Figure 4). Disturbed forest consistently registered the highest number of individuals across all years, followed by primary forest, indicating a high tolerance or adaptability of several species to moderately altered habitats. In contrast, swamp (grassland), ECAF facilities, swamp forest, and stream-slope habitats exhibited substantially lower abundances, with records remaining comparatively stable and low over time. Overall, the pattern suggests marked differences in habitat use and detectability, with forested habitats supporting higher individual abundances and contributing most strongly to the temporal variation observed in the dataset.

### 3.3. Alpha Diversity

The disturbed forest had the highest number of species (*n* = 35), while stream slope had the lowest species richness (*n* = 2). The alpha diversity of the herpetofauna species sampled at the ECAF station showed variability with patterns of decline and recovery over the six years of sampling. The highest value in the Chao-1 index was obtained in year 1 (60.53), while in the other years the value decreased to 32.98, 19.63, 22.41 and 20.12. In year 6, there was an increase in diversity, reaching 41.19. The Shannon-Weaver index showed consistently high values from year 1 to year 6: 3.223; 3.156; 2.856; 2.942; 2.402; and 3.097. Finally, the Simpson index values were close to 1 (0.9423, 0.9472, 0.9389, 0.9346, 0.8614 and 0.932), reflecting high diversity. Overall, the three indices indicate high species diversity in the area, with a decrease observed in year 5.

#### 3.3.1. Sampling Effort and Species Richness Patterns

**Figure 5:**
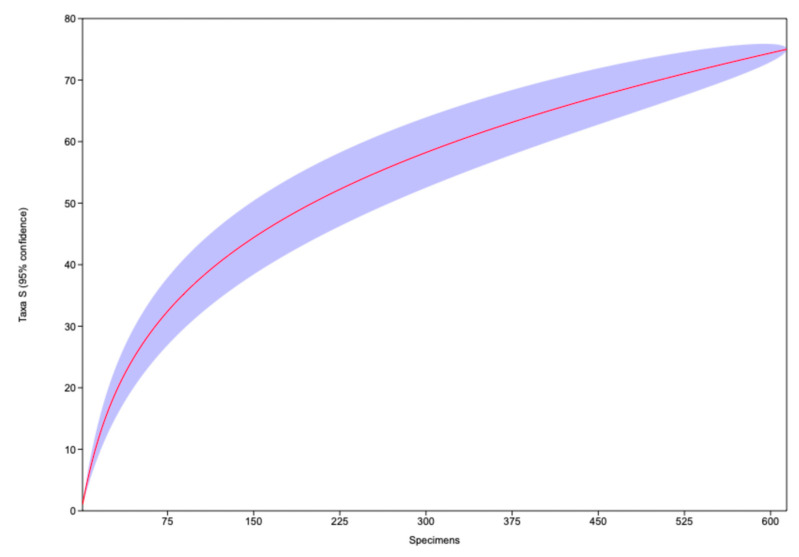
Species accumulation curve: The red line (average curve), along with its confidence interval 95% (shaded area).

The species accumulation curve (Figure 5) shows a growing increase in richness recorded during the first units of effort, followed by a trend that clearly shows an increase in the number of specimens sampled. This pattern indicates that most common species were detected in the early stages of sampling, while additional species recorded later probably correspond to taxa of low abundance or difficult to detect. However, the near-asymptotic behavior of the curve indicates that the area has been well studied. Based on the variability of species richness, only a limited number of additional species that could be likely rare, cryptic, or characterized by low detectability are expected to be recorded with increased sampling effort. This pattern underscores the importance of maintaining or supplementing sampling efforts to achieve a more complete representation of local diversity.

#### 3.3.2. Similarity of Species Between Years of Sampling

**Figure 6:**
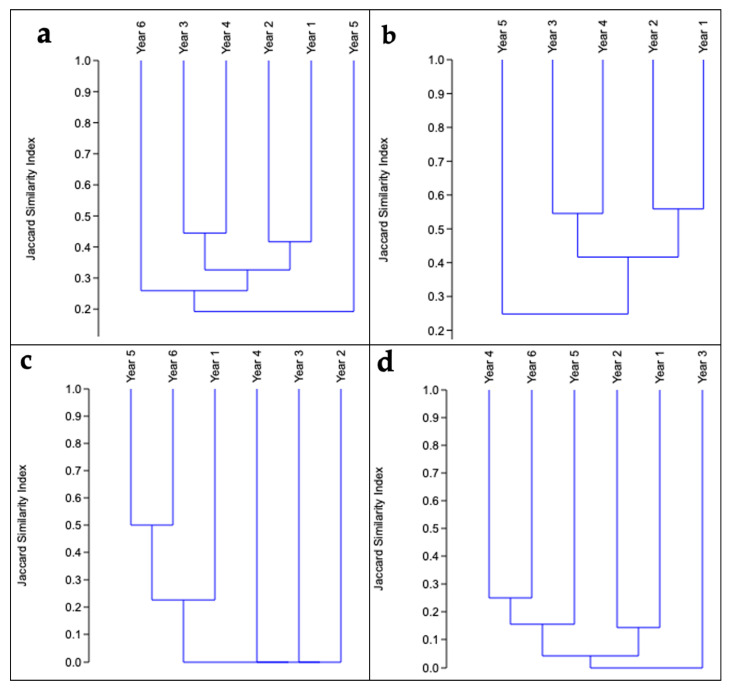
Species similarity: Jaccard index. (**a**) Overall Jaccard. (**b**) Jaccard Anura. (**c**) Jaccard Squamata (**d**) Jaccard Serpentes.

The dendrogram (Figure 6a), which is based on the Jaccard Similarity Index, shows that the composition of the herpetofauna community varied considerably over the six years of sampling. This is reflected in the low overall similarity index. The first four years, Years 3 and 4, and Years 1 and 2, have the most similar species composition (above 0.4), forming clusters at moderate similarity levels. However, all these groups are clustered together at Jaccard levels below 0.30, indicating high turnover.

The dendrogram (Figure 6b), which analyses the similarity in the species composition of Anura, shows moderate variability. The groups with the most similar composition (above 0.5) are also Years 3 and 4, and Years 1 and 2. Year 5 is the most distinct, joining the main cluster at a low index. This indicates that this period had the most unique composition of anurans in terms of species presence/absence.

The dendrogram of lizards (Figure 6c) shows extremely low temporal similarity in species composition over the six years, with a highly variable community structure. The only cluster that forms with significant similarity is that of Year 5 and Year 6, as it shows a strong connection with an index above 0.5.

The dendrogram for snakes (Figure 6d) shows an extremely low level of temporal similarity, with values below 0.3. Species composition differs greatly between years.

### 3.4. Conservation Status

Considering the number of individuals for each species, the analysis of the conservation status of registered species reveals a pattern characterized by a high number of species classified as Least Concern (LC) (*n* = 64, 85%) (Table 2). There are species in categories of greater risk such as ‘Vulnerable’ (VU) (*n* = 2), and ‘Endangered’ (EN) (*n* = 2) for 5.3%. Notably, there is one species (*Bolitoglossa peruviana*) in the ‘Data-Deficient’ (DD) category, which means there is no sufficient data available to assign a category. Eight percent of the taxa evaluated have a conservation status (NE) because they were not identified at the species level, only at the genus level. They were assigned the NE category due to taxonomic complexity and the absence of reliable diagnoses at the species level, which prevents the assignment of a formal conservation status. For this reason, none of the species has been included in a conservation status category.

## 4. Discussion

The results of the study include 85 species belonging to the order Anura, Caudata and Squamata. This confirms that the province of Pastaza is one of the most diverse areas for herpetofauna in Ecuador. Because in comparison, is relative low the number of amphibian and reptile species known from other provinces of Ecuador, for example, in Pichincha (*n* = 145 spp.), Esmeraldas (*n* = 101 spp.), Carchi (*n* = 100 spp.) and Inbabura (*n* = 96 spp.) [40].

This assertion is supported by previous estimates highlighting the Amazon as a hotspot for speciation and endemism [56,57,58]. The observed composition partially coincides with inventories carried out in areas with similar ecological dynamics to those of the Neotropics, such as Loreto in Peru [59], where 54 species and 10 families were recorded, including nine families of anurans and one family of caudate species.

The high overall coverage value indicates that the sampling effort was significant, enabling the characterization of a large proportion of the herpetofauna community in the province of Pastaza. Although the accumulation curve at the end suggests the existence of rare species that have not yet been detected, a common pattern of diverse Amazonian assembly can be observed. The differences between habitats reflect characteristics and greater complexity, resulting in greater richness and wider distribution. However, it should be noted that generalist species such as *Rhinella marina* and *Scinax ruber* dominate disturbed or anthropogenic habitats. This pattern is consistent with studies documenting the adaptability and tolerance of certain taxa to disturbance, and conversely, the strong dependence of others on specific habitats [60,61,62,63]. There is evidence of a community with a heterogeneous distribution of abundance, probably because the humid habitat where certain species find optimal conditions for reproduction and feeding.

Forested habitats (disturbed and primary forest) concentrated the highest number of individual records throughout the study period, whereas open or specialized habitats showed consistently lower abundances. This pattern suggests a stronger association of the herpetofaunal assemblage with forest environments, including disturbed areas, and highlights the relative importance of forest cover in maintaining high levels of individual occurrence across years.

The temporal variation observed in alpha diversity could reveal contrasting ecological patterns, as the biology and differential sensitivity of amphibians and reptiles must be considered. reptiles [64,65]. The decline in diversity observed in the fifth year may be consistent with the sensitivity of these two groups to environmental variables, such as drought, climate change or hydrological alterations [66], as well as anthropogenic factors [67]. For instance, the limited number of salamander species found may be attributed to their high sensitivity to minimal climate and leaf litter changes [68,69]. In contrast, reptiles, being less dependent on humidity and more tolerant of temperature variations, may be more resilient to moderate disturbances [70,71]. However, lizards can be sensitive to changes in habitat structure, vegetation cover or localised anthropogenic pressure [72,73]. Snakes, on the other hand, may be less detectable due to their behaviour [74]. Data from the Chao-1 estimator show a progressive decline, followed by a recovery towards the end of the period. This indicates that the presence of rare or low-visibility species is highly variable and could be affected by random environmental factors [75,76]. The decline observed in the fifth year highlights the need for conservation strategies, such as the designation of protected areas, that consider the sensitivity of different species [77,78].

Temporal variation in Jaccard similarity analyses suggests high species turnover between years, probably reflecting annual environmental fluctuations, hydrological dynamics, changes in shelter availability and ecological community variation. This behavior is particularly evident in snakes and lizards, groups that have long been recognized for their low detection rate and high sensitivity to environmental changes Kleemann [79,80]. The similarity between the areas suggests that they have undergone similar biogeographic processes, resulting in community structures dominated by families that are widely distributed throughout the Amazon basin. These families include Hylidae, Leptodactylidae and Strabomantidae. Similarly, two species of Plethodontidae were recorded in this study area, which is consistent with previous findings [59]. The results obtained for reptiles reflected 23 species, 16 genera and eight families of the order Squamata. In a similar study conducted in the Yanayacu ravine—Itaya River in Loreto, Peru, a total of 36 species were obtained. The reptile families with the highest density that were identified in this study were Gymnophthalmidae [59]. In the northern region of Loreto, Peru, 39 reptile species were recorded, with the best-represented families being Gymnophthalmidae, Colubridae and Polychrotidae [81].

In terms of conservation status, while most species are categorized as Least Concern (LC), the presence of taxa classified as Vulnerable (VU) and Endangered (EN), particularly within sensitive genera such as *Atelopus* and *Hyloscirtus*, highlights the region’s susceptibility to various threats, including habitat loss, emerging diseases such as *Batrachochytrium dendrobatidis* [82,83], and climate change [84]. These findings align with the global amphibian decline trends reported by Luedtke, Chanson, Neam, Hobin, Maciel, Catenazzi and Stuart [5]. Amphibians and reptiles are important consumers in Neotropical ecosystems, yet they are the most threatened group of terrestrial vertebrates [85]. Consequently, reports of catastrophic declines and extinctions of amphibian species in South America have increased in recent decades [86]. There is an urgent need to strengthen monitoring and active conservation programs. Species with Data-Deficient (DD) status represent critical information gaps that could conceal undetected risks in a context of high anthropogenic pressure [87].

Although localized landuse changes have occurred in parts of Pastaza since 2018, much of the province still has extensive forest cover. The results presented should therefore be interpreted as a baseline characterization of herpetofaunal diversity. Such baseline data are essential for detecting future changes in species composition, dominance patterns, and conservation status, and present the importance of continued long-term monitoring in this highly biodiverse region. However, the future resampling of the same habitats using comparable methodologies will be essential to evaluate temporal changes in herpetofauna communities and to inform adaptive conservation strategies in the Ecuadorian Amazon.

## 5. Conclusions

The sampling effort conducted provided an adequate record of local species richness and revealed clear variations in diversity and abundance across habitats. Primary forests and riparian areas emerged as key environments for the persistence of specialised species, while the warm, humid conditions in the Pastaza region support one of Ecuador’s most diverse herpetological communities. Generating scientific information in this context is essential for understanding the region’s biological composition and for guiding conservation and sustainable-use initiatives. This is particularly important given that human-driven landscape transformation has favoured the expansion of secondary forests, plantations and grasslands.

The presence of species classified as vulnerable, endangered or data-deficient highlights the urgent need to implement conservation strategies that protect critical habitats and reduce anthropogenic pressures. Furthermore, the temporal variability observed in species composition emphasises the importance of continuous monitoring programmes, as even minor environmental shifts can have a significant impact on sensitive species. Overall, the findings suggest that the herpetofauna of Pastaza has high species richness, with a community structure shaped by ecological characteristics and disturbance gradients. This emphasises the need for adaptive conservation strategies that protect pristine habitats, manage disturbed areas, and support long-term monitoring, providing essential scientific evidence for informed land use planning and biodiversity management at local and regional scales.

## Figures and Tables

**Table 1:** GCE (Global coverage estimates).

Habitat	Coverage Estimate
Global coverage estimate	97.4%
Swamp (forest)	100%
Swamp (grassland)	97.6%
ECAF facilities	93.8%
Primary forest	96.1%
Disturbed forest	98.5%
Stream-slope	100%

**Table 2:** Individual IUCN conservation status of species.

Taxa	Conservation Status					Total
	DD	EN	LC	VU	NE	
**AMPHIBIA**						
**ANURA ORDER**						
**FAMILY AROMOBATIDAE**						
*Allobates fratisenescus*				13		13
*Allobates insperatus*			4			4
*Ameerega parvula*			9			9
**BUFONIDAE FAMILY**						
*Amazophrynella siona*			4			4
*Atelopus spumarius*			4			4
*Rhinella dapsilis*			1			1
*Rhinella festae*			8			8
*Rhinella margaritifera*			1			1
*Rhinella marina*			22			22
**CRAUGASTORIDAE FAMILY**						
*Niceforonia nigrovittata*			1			1
*Noblella lochites*		1				1
*Oreobates quixensis*			1			1
*Pristimantis altamazonicus*			45			45
*Pristimantis aureolineatus*			1			1
*Pristimantis brevicrus*			1			1
*Pristimantis carvalhoi*			12			12
*Pristimantis cremnobates*		2				2
*Pristimantis croceoinguinis*			4			4
*Pristimantis diadematus*			6			6
*Pristimantis incomptus*			9			9
*Pristimantis katoptroides*			2			2
*Pristimantis kichwarum*			8			8
*Pristimantis lacrimosus*			19			19
*Pristimantis lanthanites*			11			11
*Pristimantis luscombei*			10			10
*Pristimantis martiae*			15			15
*Pristimantis nigrogriseus*				13		13
*Pristimantis quaquaversus*			41			41
*Pristimantis trachyblepharis*			31			31
*Pristimantis* sp.					18	18
*Pristimantis* sp.					3	3
*Pristimantis* sp.					3	3
*Pristimantis* sp.					1	1
*Pristimantis* sp.					1	1
**HYLIDAE FAMILY**						
*Boana boans*			1			1
*Boana cinerascens*			1			1
*Boana lanciformis*			15			15
*Dendropsophus bifurcus*			5			5
*Dendropsophus parviceps*			33			33
*Dendropsophus rhodopeplus*			1			1
*Dendropsophus sarayacuensis*			51			51
*Hyloscirtus phyllognathus*			2			2
*Osteocephalus fuscifacies*			4			4
*Scinax funereus*			2			2
*Scinax garbei*			1			1
*Scinax ruber*			42			42
**LEPTODACTYLIDAE FAMILY**						
*Adenomera andreae*			1			1
*Adenomera hylaedactyla*			20			20
*Engystomops petersi*			36			36
*Leptodactylus discodactylus*			2			2
*Lithodytes lineatus*			9			9
**CAUDATA ORDER**						
**PLETHODONTIDAE FAMILY**						
*Bolitoglossa ecuatoriana*			1			1
*Bolitoglossa peruviana*	1					1
**REPTILIA**						
**ORDEN SQUAMATA-SAURIA**						
**DACTYLOIDAE FAMILY**						
*Anolis fuscoauratus*			13			13
*Anolis ortonii*			2			2
**GEKKONIDAE FAMILY**						
*Gonatodes caudiscutatus*			5			5
*Gonatodes concinnatus*			4			4
**GYMNOPHTHALMIDAE FAMILY**						
*Cercosaura argulus*			3			3
*Loxopholis parietalis*			7			7
*Potamites* sp.					2	2
*Potamites strangulatus*			3			3
**ORDEN SQUAMATA-SERPIENTES**						
**BOIDAE FAMILY**						
*Epicrates cenchria*			1			1
**DIPSADIDAE FAMILY**						
*Atractus elaps*			3			3
*Atractus major*			1			1
*Atractus orcesi*			1			1
*Chironius monticola*			1			1
*Drepanoides anomalus*			1			1
*Erythrolamprus festae*			2			2
*Oxyrhopus occipitalis*			1			1
*Siphlophis cervinus*			1			1
*Siphlophis compressus*			1			1
**ELAPIDAE FAMILY**						
*Micrurus narduccii*			1			1
**VIPERIDAE FAMILY**						
*Bothrocophias microphthalmus*			3			3
*Bothrops atrox*			4			4
*Bothrops taeniatus*			1			1
**Total**	**1**	**3**	**55**	**26**	**28**	**614**

## Data Availability

All data will be available upon sending a request to the authors.

## Abbreviations

The following abbreviations are used in this manuscript:

IUCN	International Union for Conservation of Nature
INAHMI	National Institute of Hydrology and Meteorology of Ecuador
ECAF	Fátima Amazonian Scientific Station
GCE	Global coverage estimator
UPGMA	Unweighted Pair Group Method using the Arithmetic Mean
LC	Least concern
VU	Vulnerable
EN	Endangered
DD	Data-deficient
NE	Not evaluated
